# Development of Stable, Maleimide-Functionalized Peptidoliposomes Against SARS-CoV-2

**DOI:** 10.3390/ijms26041629

**Published:** 2025-02-14

**Authors:** Olga Michel, Aleksandra Kaczorowska, Lucyna Matusewicz, Kliwia Piórkowska, Marlena Golec, Wiktoria Fus, Kazimierz Kuliczkowski, Aleksander F. Sikorski, Aleksander Czogalla

**Affiliations:** 1 Department of Cytobiochemistry, Faculty of Biotechnology, University of Wrocław, F. Joliot Curie 14a, 50-383 Wrocław, Poland; 2 Department of Biomedical Engineering, Faculty of Fundamental Problems of Technology, Wrocław University of Science and Technology, Wybrzeże S. Wyspiańskiego 27, 50-370 Wrocław, Poland; 3 Silesian Park of Medical Technology Kardio-Med Silesia, M. Curie-Skłodowskiej 10C, 41-800 Zabrze, Poland; 4 Acellmed Ltd., M. Curie-Skłodowskiej 10C, 41-800 Zabrze, Poland; 5 Research and Development Center, Regional Specialist Hospital, Kamienskiego 73a, 51-154 Wrocław, Poland

**Keywords:** decoy receptors, liposomes, extrusion, microfluidization, high-pressure homogenization, SARS-CoV-2, maleimide, peptidoliposomes

## Abstract

Throughout the last 5 years, extensive research has been carried out towards the development of effective treatments for coronavirus disease 2019 (COVID-19). Regardless of the worldwide efforts, only a few drugs have passed clinical trials, and there is still a need to develop therapies, especially for those who are particularly vulnerable to a severe disease course. Maleimide-functionalized liposomes are proposed to serve as a platform for the immobilization, stabilization, and delivery of a short peptide sequence with high affinity towards severe acute respiratory syndrome coronavirus 2 (SARS-CoV-2). However, extensive optimizations should be performed in order to achieve features required for a reliable drug candidate, such as homogeneity of physical parameters and their long-term stability. Here, we present a step-by-step development process for maleimide-functionalized liposomes, which—once decorated with the SARS-CoV-2-binding peptide—could inhibit the infection progress of COVID-19. The main emphasis is placed on defining optimal lipid composition and formation conditions of PEGylated liposomes. We propose that the developed nanocarrier technology can be used as a universal platform for the construction of multiple antiviral agents.

## 1. Introduction

The World Health Organization (WHO) declared the outbreak of the coronavirus disease 2019 (COVID-19) pandemic on 11 March 2020. Since then, over 7,000,000 COVID-19-related deaths have been reported worldwide [1], making causative severe acute respiratory syndrome coronavirus 2 (SARS-CoV-2) one of the deadliest viruses in human history. Given the rapid spread and severity of symptoms, increased efforts have been made to develop drugs against this novel viral infection, resulting in more than 10,000 clinical trials registered for COVID-19 since the pandemic’s outbreak [2,3]. However, so far, only several drugs for COVID-19 have been approved or are under Emergency Use Authorization (EUA) by the U.S. Food and Drug Administration (FDA) [4]. These can be divided into four groups: (i) antiviral agents, (ii) immune modulators, (iii) monoclonal antibodies (mAbs) targeting SARS-CoV-2, and (iv) renal replacement therapy solutions. The first group includes drugs that reduce the risk of hospitalization or death—Paxlovid (nirmatrelvir and ritonavir) and Lagevrio (molnupiravir), as well as Veklury (remdesivir), which is also approved to treat or support patients hospitalized due to COVID-19. The primary role of immune modulators is to help suppress the hyperinflammation frequently accompanying SARS-CoV-2 infection. This group currently includes Actemra (tocilizumab), Olumiant (baricitinib), Kineret (anakinra), and Gohibic (vilobelimab). Among the anti-SARS-CoV-2 monoclonal antibodies, Bebtelovimab, Sotrovimab, and REGEN-COV (Casirivimab and Imdevimab) were, at one point, the only mAb-based drugs for the treatment of COVID-19. However, all of them were withdrawn from use due to the high-frequency appearances of non-susceptible SARS-CoV-2 variants. The long-circulating COVID-19-preventive mAb drug, i.e., Evusheld (tixagevimab co-packaged with cilgavimab), met the same fate; however, it was replaced by Pemgarda, which currently remains the only pre-exposure prophylaxis drug for high-risk patients. The last group of drugs is dedicated to patients treated with continuous renal replacement therapy, and it is represented by Regiocit and Fresenius Medical multiFiltrate/multiBic/multiPlus replacement solutions—both authorized under EUA for a certain group of adult patients. The limited number of available drugs and the withdrawal of those released on the market reflect the challenges in the development process of anti-SARS-CoV-2 drugs that are highly effective yet resistant to frequent virus mutations. At the same time, it is commonly believed that the next pandemic is a matter of ‘when’, not ‘if’ [5,6,7]. Developing a universal, antiviral platform is therefore not only an important step towards the enhanced treatment of COVID-19 disease but can also increase our level of preparedness for future pandemics.

Understanding and modifying the immune response to SARS-CoV-2 infection became a matter of utmost importance as the pandemic progressed. The vaccine’s development was predominantly focused on the viral spike (S) glycoprotein [8], which plays a key role in virus entry through its interaction with the angiotensin-converting enzyme 2 (ACE2) receptor at host cell surfaces [9,10]. As soon as it was demonstrated that SARS-CoV-2 polyclonal antibodies inhibit SARS-CoV-2 spike-mediated cell penetration [10], the concept emerged to deliver the genetic information to produce the antigen in the form of nucleic acid-based messenger RNA (mRNA) [11]. Unfortunately, the utility of the mRNA vectors is majorly limited by their instability in the human body, as well as their high immunogenicity [12]. The entrapment of mRNA inside the PEGylated liposomes opened new doors for the possibility of adapting to the evolution of the virus [13], but also became a prime example of how mature vesicle technology can be successfully employed to face the SARS-CoV-2 infection. One possible approach to antiviral therapy is multiple applications of decoy receptors using various native or recombinant formulations of ACE2 or peptides derived from the native sequence (for recent reviews see e.g., Li J et al. (2024) [14], Zhang et al. (2023) [15], or Matusewicz et al. (2022) [2]). One of the biggest problems in using recombinant ACE2 or peptides simulating receptor sequences is their formulation, which needs to allow for proper delivery and effective inhibition of the virus–host cell interaction. Our aim is to construct a decoy receptor liposomal carrier that would ensure effective inhibition of virus interaction with cells bearing the ACE2 receptor and be suitable for delivery via nebulization. However, the production of a liposome-based drug requires significant optimization. Besides expressing well-documented functionality, liposomes must be characterized by excellent homogeneity and long-term stability. One way to enhance both the functionality and stability of liposomes is modifying the liposome surface with maleimide. It was demonstrated that employing as little as 0.3 mol% of maleimide into liposomes led to faster internalization and protruded liposomes’ presence at the injection site, resulting in the remarkedly improved drug delivery efficiency of liposomes in vitro and in vivo [16]. In-depth studies have revealed that thiol groups facilitate the cellular uptake of maleimide-modified liposomes and that the interaction of maleimide with thiols induces alternative liposome internalization, including endocytosis and energy-independent transport [17]. The therapeutic potential of maleimide-functionalized liposomes has been revealed in several anticancer studies in which enhanced penetration, prolonged retention, and decreased toxicity were achieved in vitro and in vivo [18,19]. These encouraging results indicate that free maleimide groups on liposome surfaces may boost their biological effects. Apart from the entrapment of cytotoxic agents, maleimide-functionalized liposomes have been used in anticancer treatment to carry targeting molecules, such as p53-directed LinTT1 peptide [20], antibodies [21,22] or aptamers [23,24], to name a few. A similar approach is employed in the present study; however, instead of a targeting molecule, liposomes are decorated with a therapeutic agent, a short peptide sequence with high affinity towards the receptor-binding domain (RBD) of the SARS-CoV-2 S protein. The main goal of this study is to prepare and characterize a stable formulation based on small lipid vesicles (liposomes) decorated with human ACE2-derived peptides that can be used for further functionality studies. The greatest emphasis is placed on optimizing the lipid composition and the calibration process (and their impact on the preparation’s stability), as well as the process of peptide attachment to vesicles via maleimide residues.

## 2. Results and Discussion

The results presented here include studies conducted on 51 preparations with 19 different lipid compositions, as shown below (Table 1).

Right after formulation, vesicles were calibrated. Next, a portion of the liposomes were retained for further studies (such as phosphorus concentration measurements and, in some cases, dynamic light scattering, DLS, studies) and the rest was conjugated with the peptide. Finally, the excess peptide was removed through dialysis to receive the final product, referred to as “peptidoliposomes”. The complete procedure for the preparation of the peptidoliposomes is described and graphically presented in the Section 3.3. The optimization of peptidoliposome production included the selection of the liposome calibration method (pressure extrusion vs. high-pressure homogenization), the selection of the most favorable lipid composition, and the determination of the optimal conditions for peptide attachment to liposomes.

### 2.1. Selection of Vesicle Calibration Method

#### 2.1.1. The Impact of the High-Pressure Homogenization (HPH) Chamber on the Liposome Calibration Process Performed with the Microfluidics LM20 System

**Figure 1:**
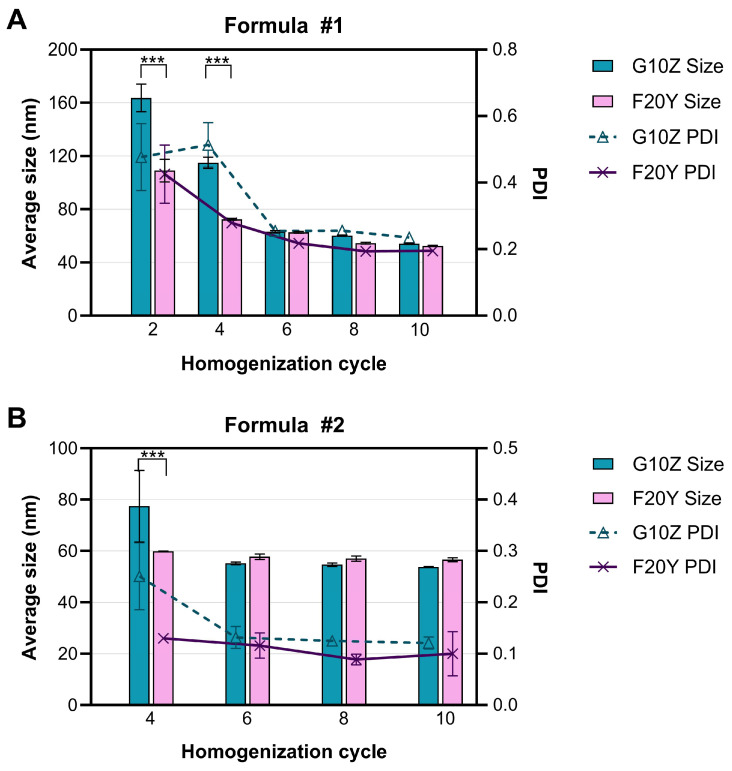
The impact of a high-pressure homogenization chamber (G10Z and F20Y) on the course of the homogenization process at 22,500 psi, as monitored via the size distribution of liposomes composed of HSPC/CHOL (**A**) or HSPC/CHOL/DSPC/POPG/DSPE-PEG_1000_ and DSPE-PEG_2000_-Mal (**B**); *** *p* ≤ 0.001.

In the project, two techniques were employed for liposome calibration: pressure extrusion and high-pressure homogenization (HPH). Both techniques are widely acknowledged for liposome production [25,26,27,28,29,30]. For HPH, two Microfluidics LM20 systems were used with different chambers (G10Z vs. F20Y, both provided by Microfluidics). The influence of the microfluidic homogenization chamber on the calibration process was examined in liposomes composed of HSPC/CHOL (Formula #1) and HSPC/CHOL/DSPC/POPG/DSPE-PEG_1000_/DSPE-PEG_2000_-Mal (Formula #2). Regardless of the lipid composition, an equilibrium in the hydrodynamic diameter of liposomes was achieved more quickly (with fewer cycles) with a liposome-dedicated chamber, F20Y, than with the universal chamber, G10Z (Figure 1). On the other hand, significant differences were noted only up to the fourth cycle, implying that the impact of the homogenization chamber on liposome calibration diminishes with an increasing number of homogenization cycles. The final average hydrodynamic diameter of liposomes calibrated with the G10Z chamber was 54.16 (±0.62) nm for Formula #1 (Figure 1A) and 53.81 (±0.22) nm for Formula #2 (Figure 1B). Correspondingly, the diameter of liposomes calibrated with the F20Y chamber was 52.54 (±0.32) nm and 56.61 (±0.74) nm for Formula #1 and #2, respectively. After the last homogenization cycle, liposomes calibrated with the F20Y chamber were characterized by generally lower values of the polydispersity index (PDI) compared to liposomes produced with the G10Z chamber; nevertheless, the differences were not statistically significant (*p* > 0.05).

Previous studies on food nanoemulsions show that the appropriate geometry of the homogenization chamber may support obtaining uniform fluid dynamic conditions, which ensure a narrow droplet size distribution [31]. However, as the microfluidizer is usually equipped with a specific type of chamber, the data on the impact of chamber geometry on nanoemulsions are limited. In a study by Talsma et al. (1989), the M110 HPH apparatus was used with two chambers to produce liposomes (chambers A and B) [25]. The results are consistent with our study, as the impact of the chamber on liposome size was limited and was reduced by the increased number of homogenization cycles. However, chamber geometry was never discussed in the above-mentioned study. In the presented project, two identical LM 20 Microfluidizers were used, each with a different homogenization chamber. G10Z is a Z-type chamber, meaning that the lipid suspension is shot against the wall of the reaction chamber twice, whereas in a Y-type chamber, the liquid flow is split up into two paths, which are then redirected towards each other, thereby doubling the acting shear forces [32]. To our knowledge, our study is the first to compare the outcomes of liposome calibration with Y- and Z-type chambers. For both tested lipid compositions, liposomes subjected from six up to ten homogenization cycles were similar in terms of size and homogeneity. However, the equilibrium was reached with fewer cycles for a setup with an F20Y chamber. Since emulsion over-processing with HPH is possible, leading to phase separation and loss of stability [33], it is favorable to minimize the number of homogenization cycles. Based on those findings, a homogenizer equipped with an F20Y chamber was selected for further studies.

#### 2.1.2. The Impact of the Calibration on the Long-Term Stability of Peptidoliposomes

**Figure 2:**
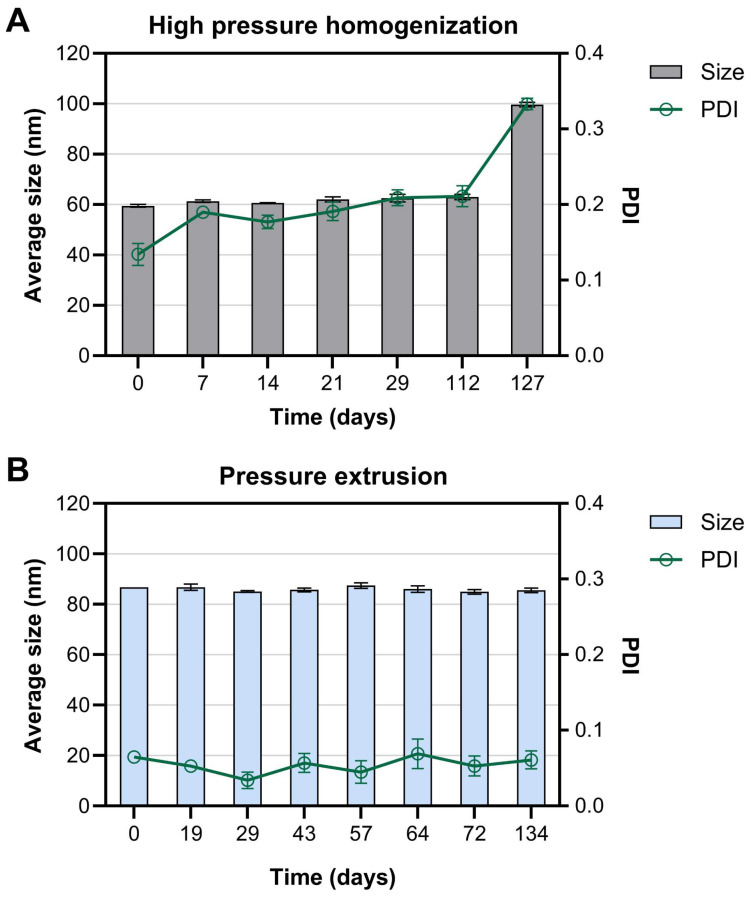
The influence of the calibration method on the long-term stability of peptidoliposomes (Formula #3). (**A**) Calibration with the high-pressure homogenization using the LM20 microfluidizer device; (**B**) calibration with pressure extrusion technique.

As the designed liposomes are intended to serve as a platform for a peptide-based SARS-CoV-2-neutralising system, they must be stable upon storage (most preferably at room temperature or at 4 °C) for at least a couple of months; therefore, long-term stability is one of the most crucial parameters for quality assessment. To determine the optimal calibration conditions in terms of product stability, liposomes consisting of HSPC/CHOL/DSPC/DPPG/DSPE-PEG_1000_/DSPE-PEG_2000_-Mal (Formula #3) were subjected to calibration either by HPH with Microfluidizer LM20 equipped with an F20Y chamber or by the pressure extrusion technique. Both techniques enabled the formation of stable and homogeneous peptidoliposomes (Figure 2).

With the HPH method, smaller peptidoliposomes were produced, with an average hydrodynamic diameter of 59.46 nm (compared to 86.72 nm in their extruded counterparts). On the other hand, the suspension after homogenization was less homogeneous, which was reflected by a higher PDI (0.134 versus 0.065 in the extruded preparation). Homogenized peptidoliposomes remained stable for around 3 months at 4 °C, and afterward, the polydispersity significantly increased (Figure 2A). On the contrary, preparation based on liposomes subjected to pressure extrusion maintained high homogeneity for at least 134 days (Figure 2B), leading to the conclusion that pressure extrusion is superior to HPH in terms of peptidoliposome stability and polydispersity. This observation agrees with other studies with various lipid compositions [25,29]. On the other hand, in the study on adenovirus-containing liposomes, preparations produced with HPH and extrusion had similar performance in terms of transduction efficiencies, physicochemical characterization, and long-term storage stability [34], showing that the effects of calibration may vary depending on liposomes’ composition.

#### 2.1.3. The Impact of the Calibration Method on Lipid Loss

Pressure extrusion has been successfully used to produce liposomes for decades [35]. Nevertheless, its use is limited by the lipid concentration, which—once too high—can lead to membrane clogging, slowing down the process and putting the product’s sterility at risk when the membrane must be replaced [34,35,36]. As a consequence, this technique is usually employed for small, lab-scale liposome syntheses. On the contrary, HPH is a high-throughput method designed for the production of nanoemulsions, liposomes, and other nanoparticles on a (semi)-industrial scale in compliance with Good Manufacturing Practice (GMP) standards [34,37,38,39,40]. The remaining issue is that homogenization requires more optimizations and may significantly reduce the lipid concentration of processed liposomes [25]. To test the effect of the calibration technique on the effective lipid concentration, liposomes with various lipid compositions (Formula #2 and Formula #3) and the same starting lipid concentration of 10 mg/mL were subjected to calibration with pressure extrusion or HPH (equipped with a F20Y chamber) before the physicochemical characteristics of the liposomes were determined.

**Figure 3:**
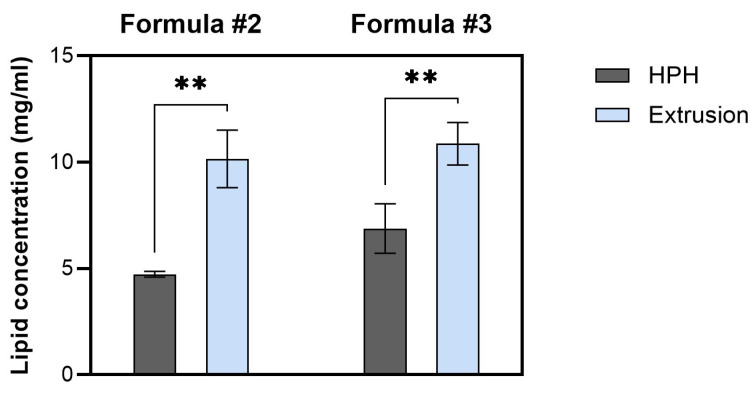
The influence of the calibration method (HPH versus pressure extrusion) on the lipid concentration of liposomes (Formulas #2 and #3); ** *p* ≤ 0.005.

Regardless of the lipid composition, liposomes calibrated with pressure extrusion had significantly higher lipid concentrations than their homogenized counterparts (Figure 3). It should be emphasized that the drop in lipid concentration of homogenized liposomes results from liposome dilution with a void volume of the LM20 Microfluidizer device (~14 mL) and not from the material loss in the calibration process. In theory, it would have been possible to retrieve almost the entire sample from the device; however, the last homogenized portions were not used in the study, due to the increased risk of air entering the system, which could have compromised the liposomes’ performance. It is worth noting that this is relevant only to low-scale preparations, as the larger the starting volume, the more negligible the dilution effect becomes.

#### 2.1.4. The Impact of the Calibration Method on the Functionality of Peptidoliposomes

**Figure 4:**
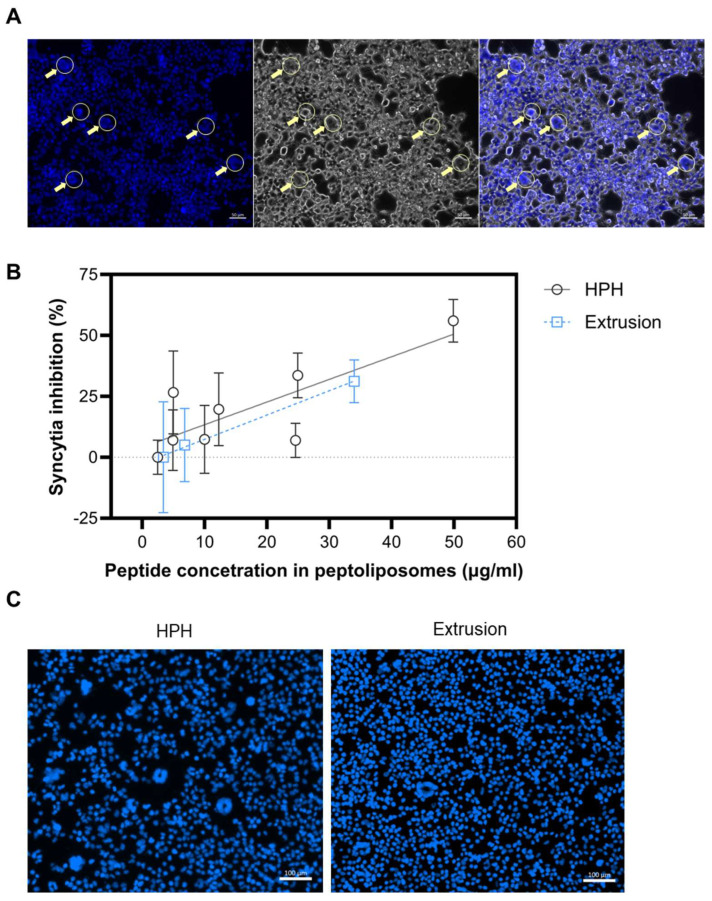
The influence of the calibration method (HPH versus pressure extrusion) on syncytia inhibition. (**A**) Syncytia forming upon the interaction of HEK-hACE2 with 293-SARS2-S, observed under a fluorescent microscope (Hoechst staining) and light microscope. Syncytia are marked with arrows, and the scalebar is 50 µm. (**B**) syncytia inhibition by anti-SARS-CoV-2 peptidoliposomes (Formula #3). Data are presented as single data points (with SD) along with lines from the simple linear regression analysis. (**C**) Representative images of syncytia formation by peptidoliposomes (Formula #3) calibrated with HPH or the extrusion technique. The scalebar is 100 µm.

The interaction of the S protein of the SARS-CoV-2 virus on the surface of infected cells with the neighboring cell receptors may induce the formation of giant structures, referred to as syncytia [41,42]. This phenomenon has further implications for the pathogenesis, viral dissemination, and immune evasion of many viruses, including SARS-CoV-2 [43,44]. The possibility to trigger syncytia in a manner independent of exogenous protease has been explored in the development of various anti-SARS-CoV-2 strategies, including studies on the cross-neutralization activity between convalescent sera from SARS and COVID-19 patients [41], or in the screening of fusion inhibitors [43]. In this study, the syncytia formation assay was employed to compare the functionality of peptidoliposomes consisting of HSPC/CHOL/DSPC/DPPG/DSPE-PEG_1000_/DSPE-PEG_2000_-Mal (Formula #3) calibrated with either HPH or the extrusion method. No significant differences were noted for the tested parameters (Figure 4).

### 2.2. Selection of the Optimal Lipid Composition

#### 2.2.1. Modification of Phosphatidylcholine (PC)

Both synthetic and natural phospholipids are used in the pharmaceutical industry. However, the latter are currently preferred as they are derived from renewable sources, are developed in more environmentally friendly production processes, and are available on a larger scale at relatively low costs compared to synthetic phospholipids [45]. On the other hand, synthetic lipids do not require validated purification procedures and have better batch-to-batch reproducibility. Distearoylphosphatidylcholine (DSPC) was found to enhance encapsulation efficiency and liposome stability, allowing for greater effectiveness in bioprocessing [46,47]. In this study, the impact of phosphatidylcholine (PC) modification on the stability of the peptidoliposomal formulation was verified using the DLS and zeta potential (ZP) measurements. The impact of hydro soy phosphatidylcholine (HSPC) replacement with DSPC was examined on two formulations prepared from homogenized liposomes. As confirmed by *t*-test analyses, peptidoliposomes prepared primarily from HSPC (Formula #3) had a smaller average diameter (*p* < 0.001) and PDI (*p* < 0.005) than their DSPC counterparts (Formula #4; Table 2).

**Figure 5:**
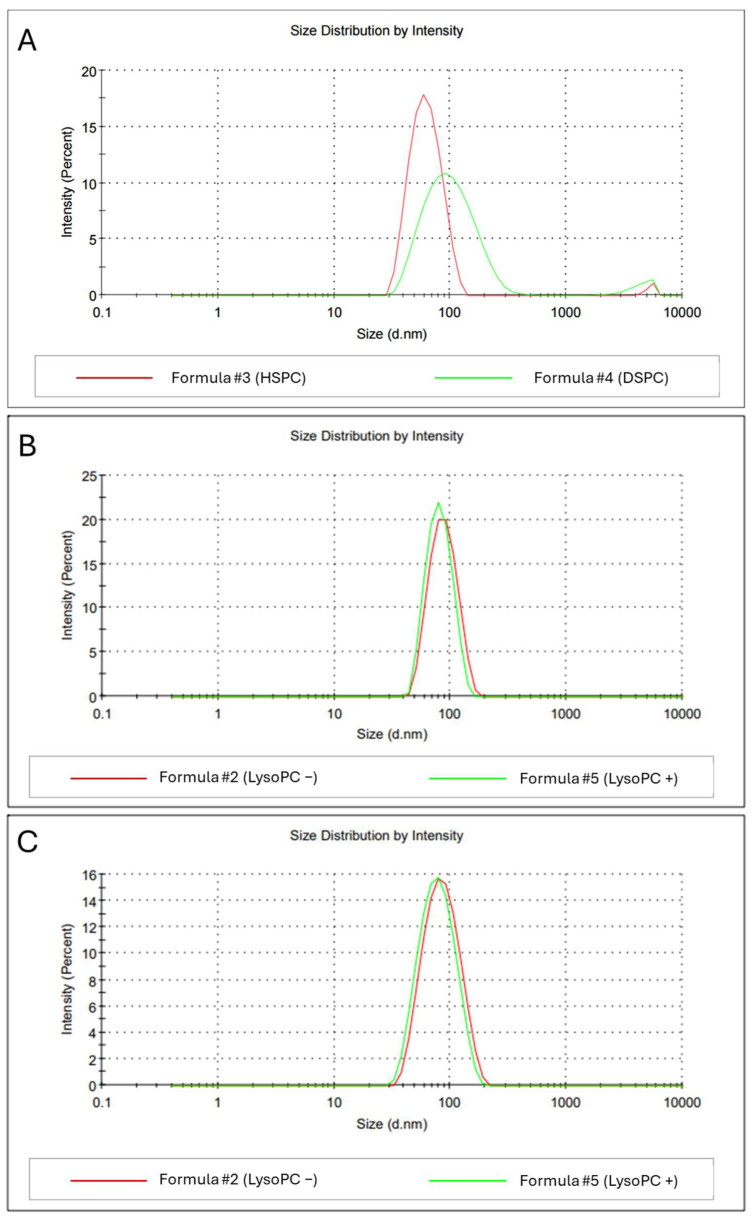
The influence of phosphatidylcholine (PC) modification on the homogeneity of anti-SARS-CoV-2 peptidoliposomes. (**A**) Average hydrodynamic diameters of preparations with the leading component being HSPC (Formula#3) or DSPC (Formula#4) after 30 days at 4 °C. (**B**,**C**) Average hydrodynamic diameters of peptidoliposomes without (Formula #2) and with the addition of LysoPC (Formula #5) after 204 days of incubation at 4 °C and after the accelerated aging assay, respectively.

The replacement of HSPC with DSPC impaired the stability of the peptidoliposomal formulation, which was reflected by the increased average hydrodynamic diameter (to 95.08 nm) and elevated PDI (0.276) after 30 days of storage at 4 °C (Figure 5).

DSPC is believed to increase the rigidity of liposomes. However, data from the molecular dynamics studies and flicker noise spectroscopy measurements on HSPC/DSPC bilayers demonstrate that the bending rigidity coefficient was slightly lower for DSPC than HSPC [48]. Nevertheless, HSPC and DSPC bilayers were characterized by similar geometry, with similar outcomes for membrane thickness and area per lipid. To verify if rigidity was a key parameter influencing the stability of tested peptidoliposomes, lysophosphatidylcholine (LysoPC) was introduced into the composition. LysoPCs can occur as minor constituents in biological membranes, where they play an essential role in phospholipid metabolism and signaling [49]. Incorporating lysolipids with a low-value molecular packing parameter (*p* < 1) increases the curvature stress in the bilayer, resulting in more leaky membranes [50,51,52]. The stability of two peptidoliposomal formulations was compared, each based on extruded liposomes—one composed of HSPC/CHOL/DSPC/POPG/DSPE-PEG_1000_/DSPE-PEG_2000_-Mal (Formula #2) and the other with 1 mol% LysoPC in lieu of HSPC (Formula #5). Peptidoliposomes with added LysoPC were slightly smaller than their HSPC counterparts (*p* < 0.001) but did not significantly differ in terms of PDI or ZP (Table 2). Both formulations with or without LysoPC were equally stable for at least 204 days of incubation at 4 °C (Figure 5B) and under the conditions of the accelerated aging assay (72 h at 37 °C in DMEM culture medium; Figure 5C). Although data do not support LysoPC inclusion in the anti-SARS-CoV-2 peptidoliposomes, the knowledge that up to 1 mol% of Lyso PC can be safely included in liposomes can be useful for further pharmacological specification and drug development to increase liposome functionality in vivo [53].

#### 2.2.2. Modification of the Cholesterol (CHOL) Concentration

Cholesterol (CHOL) is known to exert stabilizing effects when incorporated into the lipid composition of liposomes [54,55,56]. In the project, we examined the impact of increased CHOL (balanced with HSPC depletion) on the basic characteristics of peptidoliposomes as well as their stability. All preparations were manufactured from liposomes calibrated with the pressure extrusion technique. As confirmed by *t*-test analysis, peptidoliposomes with 10 mol% CHOL were slightly smaller than their counterparts with 20 mol% CHOL (*p* < 0.05) and significantly smaller than peptidoliposomes with 30 mol% CHOL (*p* < 0.001; Table 3). Whereas formulations with 10 and 20 mol% CHOL had similar polydispersity, that of the formulation with CHOL rose to 30 mol% (Formula #7), and it was significantly less homogeneous compared with those with lower cholesterol (Formula #2 and Formula #6; *p* ≤ 0.01). Changes in CHOL concentration did not affect the zeta potential of peptidoliposomes (Table 3).

**Figure 6:**
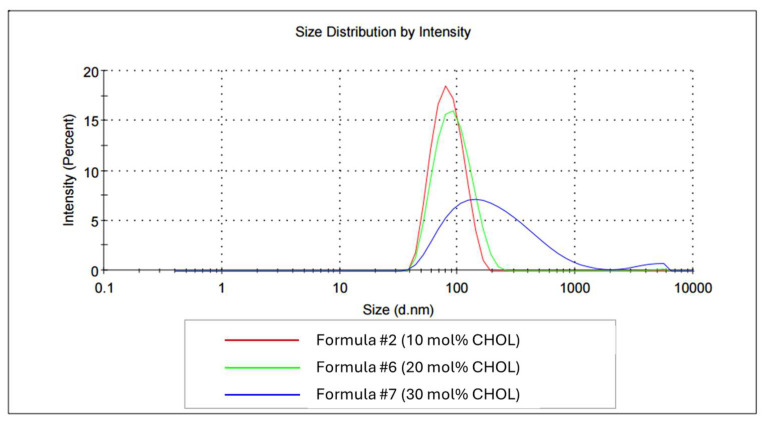
The average hydrodynamic diameters of peptidoliposomes with different cholesterol concentrations following the accelerated aging assay.

Under the conditions of the accelerated aging assay, increasing the cholesterol concentration above 10 mol% negatively affected liposome stability (Figure 6).

The average hydrodynamic diameter of liposomes with 10, 20, and 30 mol% CHOL was 78.19, 86.95, and 158.30 nm, respectively. The average PDI significantly increased with the cholesterol content reaching 0.090, 0.128, and 0.359 for peptidoliposomes with 10, 20, and 30 mol% CHOL, respectively. The increased aggregation was also reflected by the increased precipitation in the culture medium (Supplementary Material, Figure S1). It is known that the impact of cholesterol on liposome behavior may vary, as it depends on the lipid composition [57]. In membranes rich in saturated lipids, cholesterol tends to condense, increasing the order of bilayers, whereas cholesterol’s affinity towards unsaturated lipids is much lower [58]. Due to its impact on membrane fluidity, the presence of cholesterol may affect the performance of nanotherapeutics, including the encapsulation efficiency, release profile, and permeability [57,59]. In a study by Briuglia et al. (2015), the most controlled and reproducible release for drugs with different physicochemical characteristics and pharmaceutical applications was achieved for liposomes with phospholipids, with a cholesterol ratio of 2:1 [56]. In our study, such high CHOL concentrations resulted in a loss of stability, and the most favorable properties were characteristic for the preparation which contained only 10 mol% CHOL. Importantly, Matusewicz et al. (2018) demonstrated that cholesterol-free liposomes were toxic to treated cells [60], which indicates that cholesterol may be an indispensable component of the lipid composition and confirms the necessity to optimize cholesterol content during the drug development process.

#### 2.2.3. Effect of Polyethylene Glycol (PEG)-Modified Lipids

**Figure 7:**
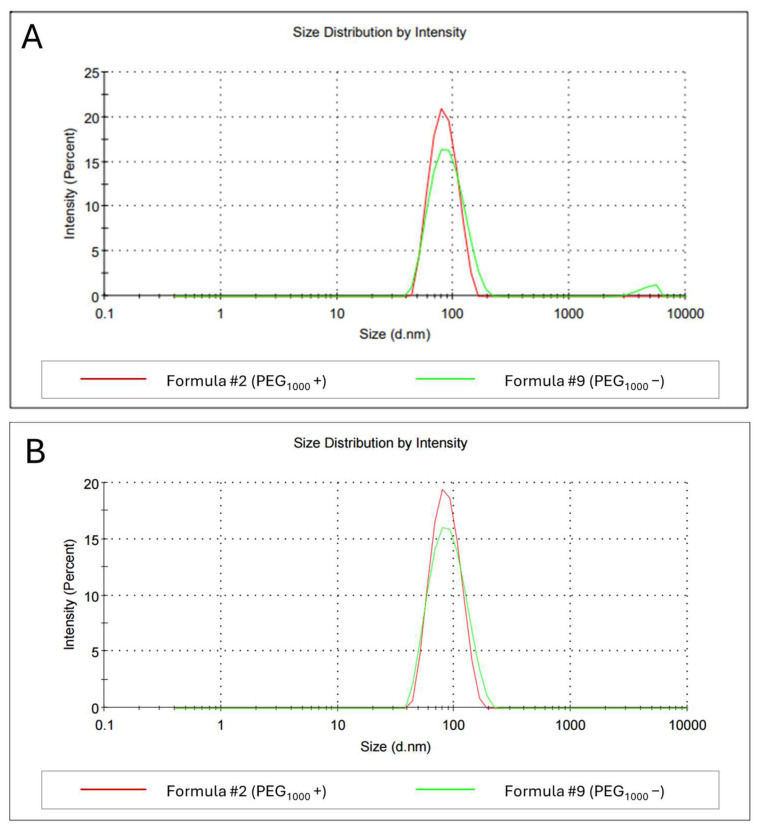
The average hydrodynamic diameters of liposomes (**A**) and peptidoliposomes (**B**) with 5 mol% PEG_1000_ (Formula #2) and without PEG^1000^ (Formula #9) after 13 days of incubation at 4 °C.

Modifications with polyethylene glycol (PEG) are known to shield liposomes from plasma proteins in the bloodstream, significantly increasing their circulation time in vivo [61,62,63]. The drug candidate developed in this study is intended for inhalation; therefore, these liposomes will be less susceptible to degradation compared to injectable formulations. However, in addition to protection against enzymes, PEG in the right proportions can provide a steric barrier for liposomes, preventing their aggregation in physiological conditions [63]. On the other hand, it has been demonstrated that PEG–lipid hydrolysis products located on the inner surface of liposomes may evoke an increase in membrane permeability; therefore, it seems vital to determine the concentration and role of PEG for each liposomal formulation individually [64]. To assess the impact of PEG on the stability of our formulation, two sets of liposomes were prepared: one containing 5 mol% PEG_1000_ (Formula #2) and the other with no PEG_1000_ (replaced by HSPC; Formula #9). Importantly, all the liposomes still contained 0.5 mol% DSPE-PEG_2000_-Mal to enable peptide attachment to the liposome surface. Liposomes and peptidoliposomes were stored at 4 °C, and the stability was tracked by DLS measurements. Significant differences were noted after 13 days of storage (Figure 7).

The depletion of PEG_1000_ had a negative impact on the homogeneity of liposomes (Figure 7A), which is reflected by the increased average hydrodynamic diameter (79.68 vs. 89.35 nm) and PDI (0.060 vs. 0.200). Notably, the impact of PEG incorporation into the lipid composition was lower for peptidoliposomes (Figure 7B) than for liposomes not decorated with peptides, suggesting that the presence of the peptide on the liposome surface had a beneficial effect on the formulation’s stability. Notwithstanding the latter, peptidoliposomes containing PEG_1000_ were still smaller (80.65 nm vs. 82.24 nm) and more homogeneous than their PEG-deprived counterparts (PDI 0.074 vs. 0.103). This was an expected result, as PEG-conjugated lipids have been known for decades to improve the blood circulation capability of liposomes [65] and have been used in various liposomal drugs, including the first FDA-approved nano-drug Doxil^®^ [66] and mRNA vaccines against SARS-CoV-2 [67]. However, the drawback of applying PEG is that immune responses have been reported after applying PEG-conjugated nanocarriers, including accelerated blood clearance and complement activation-related pseudoallergy [68,69,70]. Therefore, defining the optimal PEG concentration before functionality, toxicity, and safety studies is crucial to minimize the risk of PEG-related side effects.

#### 2.2.4. Effect of Phosphatidylglycerol (PG) Modifications

Including an anionic phospholipid, such as phosphatidylglycerol (PG), may impart a negative charge to liposomes’ surface. By enforcing electrostatic repulsion, a strong charge enhances the colloidal stability of liposomes, preventing their aggregation [71,72] Variations in PG type, such as chain length and saturation, can affect the rigidity and fluidity of liposomes [57,73,74,75]. The impact of the PG modifications was investigated in terms of the stability and functionality of the final formulation. As demonstrated in Table 4, right after synthesis, peptidoliposomes with 10 mol% dipalmitoylphosphatidylglycerol (DPPG) were of a similar size and polydispersity compared with those with 5 mol% DPPG but had a slightly lower zeta potential (*t*-test, *p* < 0.05).

**Figure 8:**
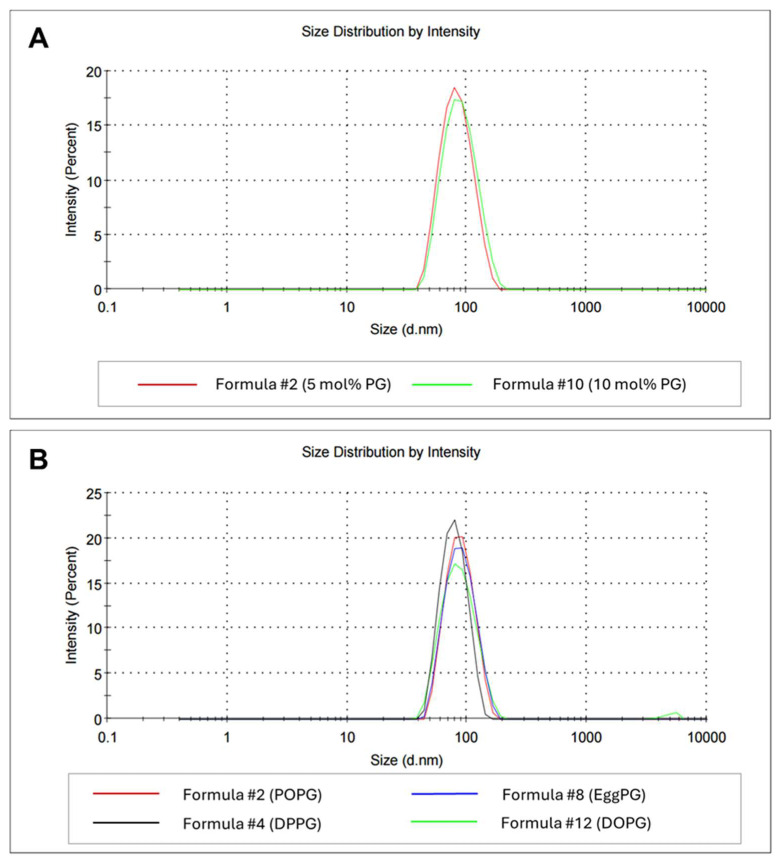
The average hydrodynamic diameters of formulations with different concentrations of POPG (**A**) and different types of PG (**B**) after 2 weeks of incubation at 4 °C. POPG—palmitoyloleoylphosphatidylglycerol; DPPG—dipalmitoylphosphatidylglycerol; EggPG—Egg L-α-phosphatidylglycerol; DOPG—dioleoylphosphatidylglycerol.

Following the two-week incubation at 4 °C, peptidoliposomes with elevated PG concentrations were characterized by slightly bigger average hydrodynamic diameters (83.55 nm vs. 79.29 nm in 5 mol% preparation) and slightly higher PDIs (0.115 vs. 0.090; Figure 8A). Within the tested PG species, the measured average hydrodynamic diameter was similar to that of all the peptidoliposomal preparations (Table 4). The homogeneity was significantly affected only with the dioleoylphosphatidylglycerol (DOPG) inclusion, which was reflected by a significantly higher PDI (*t*-test, *p* < 0.01). The differences between DOPG and other PGs intensified over time, and for DOPG peptidoliposomes, an increase in the PDI value to 0.158 was noted after 2 weeks of incubation at 4 °C (Figure 8B). Moreover, after 72 h of incubation in Dulbecco’s Modified Eagle Medium (DMEM) in the presence of 10% FBS, at 37 °C, severe precipitation of the DOPG-containing preparation was observed with a light microscope (Supplementary Material, Figure S2). Phosphatidylglycerols (PGs) seem to prevail over other negatively charged phospholipids; therefore, including them in a lipid composition can have a positive influence on liposome stability [57,74]. The ZP of all preparations was similar, indicating that the negative impact of DOPG on liposome stability cannot be attributed to the increased surface potential.

**Figure 9:**
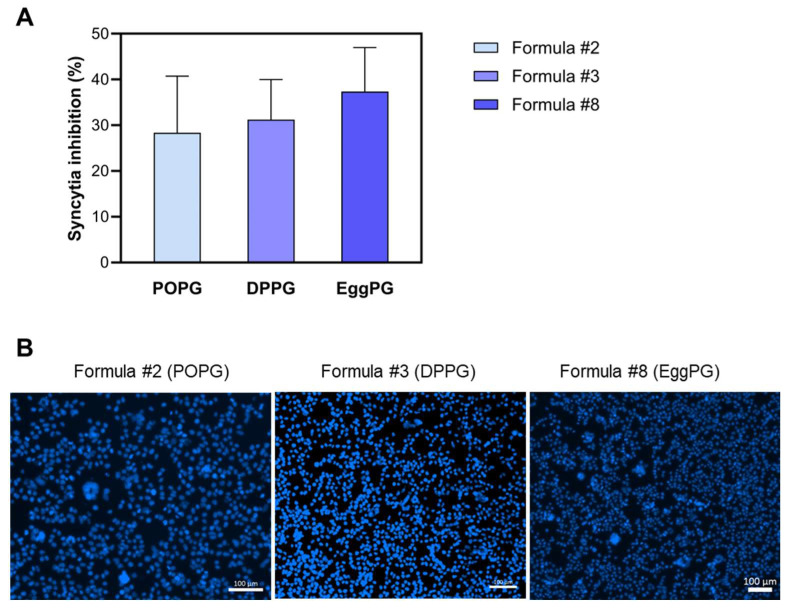
The influence of the type of PG on syncytia formation in HEK-hACE2 and 293-SARS2-S cells by anti-SARS-CoV-2 peptidoliposomes. (**A**) syncytia inhibition by peptidoliposomes containing various types of PG (Formula #2, 3 and 8) at a peptide concentration of ~35 µg/mL. (**B**) Representative images from the assay (cells stained with Hoechst). The scalebar is 100 µm. POPG—palmitoyloleoylphosphatidylglycerol; DPPG—dipalmitoylphosphatidylglycerol; EggPG—Egg L-α-phosphatidylglycerol.

As the precipitations formed in DMEM media with DOPG-containing liposomes affected the functionality assays, the following experiments were performed only with peptidoliposomes containing POPG (Formula #2), DPPG (Formula #3), and EggPG (Formula #8). As presented in Figure 9, the inhibition of syncytia formation by peptidoliposomes with various PGs was as follows: EggPG > DPPG > POPG. However, due to the high standard deviation, no statistical significance was achieved (*p* > 0.05).

EggPG-containing peptidoliposomes had satisfactory physicochemical properties and slightly better functionality compared to other PG-containing formulations. As the EggPG was also easily accessible in the GMP standard at the time of the study, this lipid was selected as a part of the nanocarrier component.

### 2.3. Defining the Optimal Conditions for Peptide Conjugation to Maleimide

#### 2.3.1. Determination of Optimal Maleimide Concentration

Maleimide is the most expensive lipid component of the studied liposomes. It was therefore crucial to determine the minimal maleimide concentration that results in satisfactory liposome coverage with the peptide but that is reasonable regarding production costs at the same time. Preparations of peptidoliposomes with various concentrations of PEG-PE maleimide derivative (0.5–20 mol%) were tested for the amount of stably bound peptide through size exclusion chromatography (SEC). The DSPE-PEG_2000_-Mal concentration was increased in exchange for DSPE-PEG_1000_ and HSPC (see Table 1 for comparison). Apart from the preparation with the highest concentration of maleimide, substitutions did not significantly influence the average hydrodynamic diameter and PDI of peptidoliposomes (Table 5).

**Figure 10:**
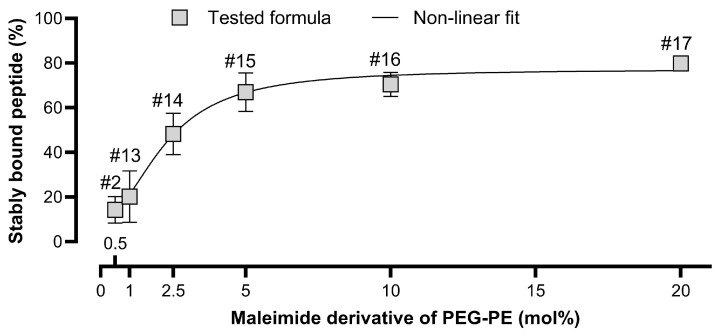
The effect of the maleimide concentration on the amount of peptide stably bound to liposomes in peptidoliposomal preparations following size exclusion chromatography (SEC). The detailed lipid composition is listed in Table 1.

Following chromatographic separation, the Brown–Forsythe and Welch ANOVA test revealed that the binding efficacy was positively influenced by the maleimide concentration only in formulations containing up to 5 mol% of the maleimide derivative of PEG-PE (Figure 10).

In contrast, no statistically significant differences were noted between formulations containing 5, 10, and 20 mol% of DSPE-PEG_2000_-Mal (*p* > 0.05). For that reason, 5 mol% DSPE-PEG_2000_-Mal was selected as the most cost-effective composition for an anti-SARS-CoV-2 liposomal drug candidate. It has to be emphasized that SEC was the only method used to determine the stably bound peptide (SBP) value; therefore, the covalent nature of the binding was not confirmed in the study. However, the peptide and liposomes must have been linked by a strong bond, since a signal from both was obtained in the same fractions. Moreover, gel filtration has been used previously to reveal the covalent nature of bonds between various particles [76,77].

#### 2.3.2. Defining the Optimal Protocol for Peptide Reduction

**Figure 11:**
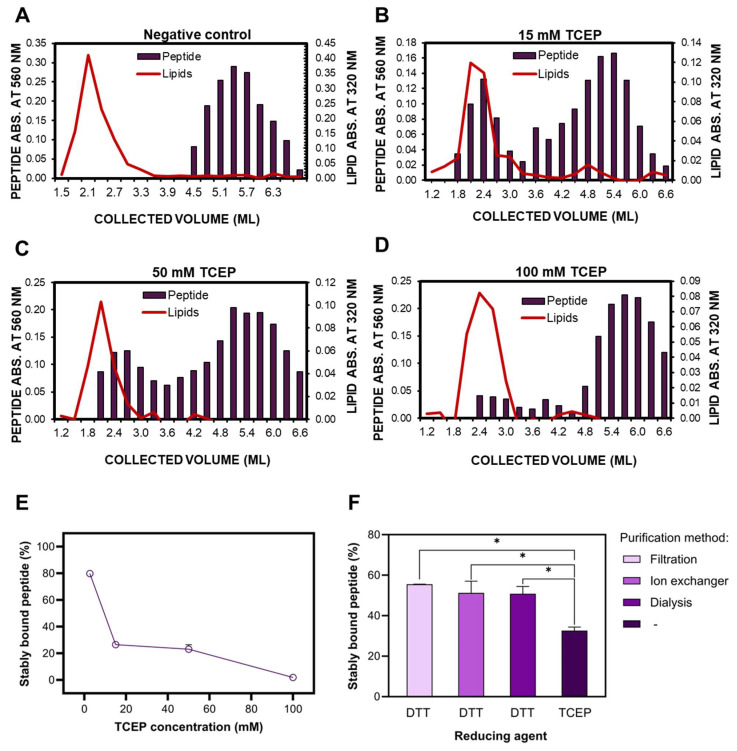
The effect of the peptide reduction on the efficacy of peptide binding to liposomes; (**A**) separation of the maleimide-deprived peptidoliposomes (Formula #18—negative control) from the unbound peptide with size exclusion chromatography (SEC); (**B**–**D**) separation of peptidoliposomes (Formula #17) from the unbound peptide with SEC following peptide reduction with 15. 50 and 100 mM TCEP; (**E**) the summary data on the TCEP concentration’s influence on peptide binding after SEC and the concentration of separated peptidoliposomes (Formula #17); (**F**) the effect of the reducing agent (DTT vs. TCEP) and the purification method (filtration, ion exchanging resin, or dialysis) on the amount of stably bound peptide after SEC and the concentration of separated peptidoliposomes (Formula #19); * *p* ≤ 0.05.

To maintain free sulfhydryl groups and to ensure the repeatability and reproducibility of the peptide attachment process, the peptide was reduced each time before conjugation to the liposomes. Tris(2-carboxyethyl)phosphine (TCEP) has been widely used to reduce disulfide bonds for over three decades. It is often preferred to other reducing agents due to its stability, wide pH range, odorless nature and easier handling (no need to remove from the reaction mixture) [78]. Here, the SEC method was employed to determine peptide binding efficacy following the reduction process (see Section 3.7). The results of our experiments may indicate that a high TCEP concentration does not favor high binding efficiency between maleimide and peptide (Figure 11A–E). This may result from TCEP’s interaction with maleimide, as TCEP had not been removed before the introduction of the ligand to the reduced peptide molecules. Several commercial sources and studies in the literature report the lack of reaction between TCEP and maleimide; therefore, the reducing agent does not have to be removed before the conjugation process [79,80,81,82]. Conversely, some studies report that TCEP is likely to react with maleimide-functionalized biomolecules under typical conditions used for bioconjugation [81,82]. Since the results of our SEC experiments favor the latter hypothesis, 1,4-Dithiothreitol (DTT) was also tested as a reducing agent. Due to its reactivity with thiol-alkylating reagents (which reduce yields of alkylation) and high toxicity [83], it is essential to remove DTT from the peptide solution before conjugation with liposomes. Three methods were used for peptide purification: filtration with centrifugation filters, ion exchanging resins, and dialysis to 50 mM HEPES/100 mM NaCl buffer. None of the methods significantly affected binding efficacy (*p* > 0.05; Figure 11F).

Regardless of the purification method, DTT reduction appeared superior to TCEP reduction in peptidoliposomes consisting of the proposed formulation (Formula #19, see Table 1). Due to its better feasibility regarding the production process and higher (but not statistically significant) binding efficacy, centrifugal ultrafiltration is a preferred method for peptide purification before conjugation to liposomes.

### 2.4. Platform Versatility and Possible Applications

Maleimide–thiol chemistry is widely used in nanocarrier production and has been demonstrated as an effective method for the development of liposomes presenting a variety of particles, including peptides [20,84,85,86], proteins [87] antibodies [21,22,88], nanobodies [84], and aptamers [23,24]. Here, we propose a platform based on a maleimide–thiol click reaction that has been optimized at different stages of production. In terms of composition, the best physicochemical parameters were achieved for Formula #19, consisting of HSPC:DSPC:CHOL:EggPG:DSPE-PEG_2000_-MAL in a molar ratio of 70:10:10:5:5. For this formula, stable and homogeneous liposomes were obtained regardless of the calibration method. When adapting the protocol, the features distinguishing liposomes obtained by either of the techniques should be taken into account. High-pressure homogenization allowed us to produce smaller liposomes, but the suspension was less homogeneous compared to the extruded counterparts (Table 6).

After a year of storage at 4 °C, in both cases, the ZP of liposomes was slightly increased. However, it did not have a significant impact on homogeneity, as the PDI remained below 0.2 for homogenized liposomes and below 0.1 for the extruded ones. The extruded peptidoliposomes were subjected to accelerated aging tests and the stability of both liposomes and peptidoliposomes was demonstrated in the presence of cell culture media with the addition of FBS at 37 °C for at least 144 h (Figure S4). The physicochemical parameters achieved for the formulation easily meet the acceptance criteria for drug delivery systems, staying far from the safety margin [89,90,91]. In addition to the liposome preparation, a conjugation reaction was optimized in terms of the peptide:maleimide ratio and conjugation protocol. In the pilot experiment, the same conditions were used to obtain liposomes conjugated with another peptide, targeting a different virus. The resulting formulations were equally stable and homogeneous, showing that similar conditions can be used to achieve successful conjugation with various molecules. However, it should be noted that the maleimide to thiol molar ratio may require modification for different conjugates to reach the optimal reaction efficiency [84]. Importantly, in the present study, liposomes were not loaded with any agents, but it is possible to use the same formula for the entrapment of various therapeutic agents. The stage at which the agent is added to the formulation should depend on the physicochemical properties and thermal stability of the agent.

## 3. Materials and Methods

### 3.1. Chemicals

The following lipids were used in the study: cholesterol (CHOL, cat. no. PA-03-0100-E), purchased from POL-AURA (Zabrze, Poland) and from Avanti (Alabaster, AL, USA); cat. no. 700000P), hydrogenated soy L-α-phosphatidylcholine (HSPC, cat. no. 840058P), 1-palmitoyl-2-oleoyl-sn-glycero-3-phosphatidylglycerol (POPG, cat. no. 840457P), 1,2-distearoyl-sn-glycero-3-phosphocholine (DSPC, cat. no. 850365C), 1,2-distearoyl-sn-glycero-3-phosphoethanolamine-N-[methoxy(polyethylene glycol)-1000] ammonium salt (DSPE-PEG_1000_, cat. no. 880720P),1,2-distearoyl-sn-glycero-3-phosphoethanolamine-N-[methoxy(polyethylene glycol)-2000] ammonium salt (DSPE-PEG_2000_; cat. no. 880120P) and 1,2-distearoyl-sn-glycero-3-phosphoethanolamine-N-[maleimide(polyethylene glycol)-2000] ammonium salt (DSPE-PEG_2000_-Mal, cat. no. 880126P), purchased from Avanti; HSPC (cat. no. 525600), 1,2-dipalmitoyl-sn-glycero-3-phospho-(1′-rac-glycerol), sodium salt (DPPG, cat. no. 67232-81-9), and L-α-phosphatidylglycerol from egg (EggPG, cat. no. 583500), purchased from Lipoid (Ludwigshafen, Germany). Organic solvents and sodium chloride were purchased from CHEMPUR (Piekary Śląskie, Poland). HEPES and Tris(2-carboxyethyl)phosphine (TCEP) were purchased from ROTH (Karlsruhe, Germany), and 1,4-Dithiothreitol (DTT) was purchased from A&A Biotechnology (Gdańsk, Poland).

### 3.2. The Anti-SARS-CoV-2 Peptide

Liposomes were designed to expose a short ACE2-based, 32-amino-acid-residue peptide (molecular formula C_164_H_241_N_43_O_54_S), with a molecular weight of 3711.03 Da, a theoretical pI = 4.654 and a net charge of z = −3.608 at pH 7.4, as calculated with the Prot pi calculator (www.protpi.ch/Calculator/ProteinTool; accessed on 8 November 2024). The sequence was taken from Huang et al. (2020) [92]. Each time before conjugation with maleimide, the peptide was reduced with TCEP or DTT. In the latter case, DTT was separated from the peptide either with centrifugation filters (MWCO = 2K, Millipore (Burlington, MA, USA), or Sartorius (Göttingen, Germany), with an ion exchanging resin (DEAE–Sephadex^®^ A-25 chloride form; Sigma (St. Louis, MO, USA), cat. no. A-25-120), or through dialysis to 50 mM HEPES/100 mM NaCl buffer (pH 7.2) using the dialysis membranes (2 kDa, Repligen, Waltham, MA, USA). Following purification from DTT, the peptide concentration was measured before conjugation to liposomes.

### 3.3. Preparation of Peptidoliposomes

**Figure 12:**
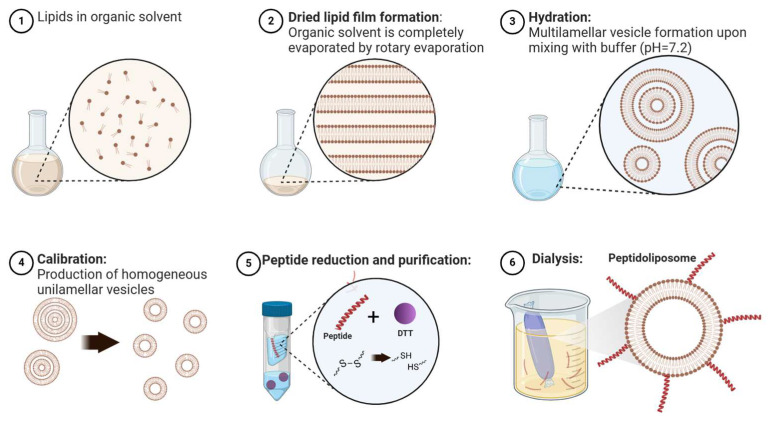
The process of synthesis of maleimide-functionalized liposomes decorated with the ACE2-derived peptide (created with BioRender and adapted and modified from Zhang et al. (2017) [93]).

Liposomes were prepared using the thin-film hydration method. Briefly, lipids were dissolved in a ratio of 9:1 in a chloroform: methanol mixture (DSPE-PEG2000-Mal) or in pure chloroform (all other lipids) and transferred in appropriate proportions into a round-bottom flask. The organic solvents were initially slowly evaporated under a nitrogen stream, and then the film was entirely dried overnight with a desiccator. Next, the lipid film was hydrated with 50 mM HEPES/100 mM NaCl buffer (pH 7.2) by gentle mixing with glass beads for 30′ at 64 °C (the number of beads was equal to half of the suspension volume in mL). After the synthesis, liposomes were calibrated through pressure extrusion or HPH. During the extrusion, nitrogen-generated pressure was used to press liposomes through track-etch polycarbonate membranes (Cytiva, Wilmington, DE, USA) in the following order: 0.2 µm (10 cycles), 0.1 µm (10 cycles), and 0.05 µm (5 cycles). Membranes were supported by a PE drain disc (Cytiva). HPH was performed with two LM20 Microfluidizer^®^ devices (Microfluidics, Westwood, MA, USA) equipped with either a F20Y or a G10Z chamber. Each time, 10 homogenization cycles were run at 22,500 psi (1551.3 bar). The liposomes were incubated with a reduced peptide for 24 h at 4 °C, and then the excess peptide was removed through dialysis to the HEPES/NaCl buffer. Dialysis was carried out with dialysis membranes (MWCO 14–50 kDa, Spectra/Por^®^) at 4 °C in two stages: first for 16 h in sample/buffer *v*/*v* 1:100, and then for another 8 h with sample/buffer *v*/*v* 1:300. A graphical sketch of the synthesis process is presented in Figure 12.

Each time after synthesis preparations were sterilized. The details on the sterilization process can be found in Appendix A.

### 3.4. Lipid Concentration Measurements

Lipid concentration was measured with the modified molybdate/ascorbic acid method first described by Murphy and Riley (1962) [94]. In brief, 50 µL of each sample of various dilutions was mixed with 0.5 mL 70% perchloric acid (CHEMPUR) and incubated for 2 h at 200 °C in glass covered vials. Next, 1 mL of ammonium molybdate (Avantor Performance Materials Poland; Gliwice, Poland) at a concentration of 25 mg/mL and 1 mL of ascorbic acid (ROTH) at a concentration of 100 mg/mL were added to each sample, and the samples were incubated for another hour at 37 °C. Next, samples were transferred on the 96-well transparent plate (200 µL per well), and the absorbance of the solutions was measured with a Rayto RT-100 microplate reader (Rayto Life and Analytical Sciences, Shenzhen, China) at λ = 750 nm. At each stage, the absorbance was compared against to the standard curve prepared from the phosphorus standard (Honeywell, Charlotte, NC, USA; cat. no. E1042-100ML).

### 3.5. Peptide Concentration Measurements

The peptide concentration in peptidoliposomes was measured with the modified bicinchoninic acid (BCA) assay, first described by Smith et al. (1985) [95]. In brief, 135 µL of each sample in various dilutions was mixed with 15 µL of 10% sodium dodecyl sulfate (SDS; Sigma) (for liposome disintegration), and 150 µL of BCA reagent (Sigma) mixed with copper (II) sulfate ((Sigma) in a proportion of 50:1) was added to each sample in a 96-well, transparent plate. Next, samples were incubated for 60 min at 37 °C, and the absorbance was measured with Promega Glomax Discover Microplate Reader (Promega, Madison, WI, USA) at λ = 560 nm. A standard curve was prepared with the freshly dissolved peptide at a concentration of 1 mg/mL.

### 3.6. Stability Measurements

Stability was assessed primarily based on changes in particle average hydrodynamics and the PDI, measured with the DLS technique. The ZP was tracked using the Dip Cell Kit (Malvern, UK). All measurements were conducted using a ZetaSizer Nano device (Malvern, UK) at 25 °C. Measurements were carried out in samples incubated for a longer duration at 4 °C or following the accelerated aging assay when samples were mixed in a ratio of 1:10 with the culture medium (DMEM. Gibco, Waltham, MA, USA) + 10% FBS and then incubated for at least 72 h at 37 °C.

### 3.7. Size Exclusion Chromatography (SEC)

Free peptides were separated from peptidoliposomes via SEC using the 5 mL columns with Sepharose^®^ CL-4B (Cytiva; column diameter to volume ratio of 1:25). A sample of 0.3 mL was transferred into a column, and fractions in volumes of 0.3 mL were collected on the 96-well transparent plate; then, the absorbance was measured at 320 nm to distinguish the liposome-containing fractions. Next, a BCA assay was employed to determine the peptide concentration in the collected fractions using two approaches: (i) to assess the separation resolution, the peptide concentration was measured directly in non-diluted fractions, and (ii) for the precise determination of the stably bound peptide, the liposome-containing fractions were concentrated with the Amicon^®^ centrifugation system (100 kDa, Millipore), and the lipid and peptide concentrations were measured in samples before and after chromatographic separation. This approach is independent of the one used for sample dilution with chromatographic columns. The amount of peptide bound to liposomes was calculated according to the following Equation (1):

SBP = (m_PEPTIDE/_m_LIPID_ after *SEC*)/(m_PEPTIDE/_m_LIPID)_ before *SEC*) × 100% (1)

where SBP stands for stably bound peptide (%), m_PEPTIDE_ is the mass of peptide per ml (µg), and m_LIPID_ is the mass of lipids (mg) per ml in the peptidoliposomal preparations.

### 3.8. Syncytia Formation Assay

A syncytia formation test was performed according to the procedure described by Lu et al. (2005) [96]. Giant, multinucleated cells were formed as a result of the fusion of two HEK293 sublines, one exposing the ACE2 protein on the cell membrane surface (HEK-hACE2 (Invivogen, catalog number: hkb-hace2, San Diego, CA, USA)) and the other exposing protein S of the SARS-CoV-2 virus (293-SARS2-S (Invivogen, cat. no. 293-cov2-s)). The test was optimized to be performed on a 24-well plate. Plates were coated 24 h before seeding the cells with a medium composed of albumin BSA (0.01 mg/mL, Sigma, cat. no. A8022), fibronectin (0.01 mg/mL. Sigma, cat. no. F2006), and collagen type I (0.03 mg/mL, Advanced BioMatrix, cat. no. 5006, Carlsbad, CA, USA). Before seeding them, the HEK-hACE2 and 293-SARS2-S cells were mixed in a 1:1 ratio using 20,000 cells of each line, and then incubated without or with the liposome preparation at 37 °C for 120 min. Following seeding, the cultures were incubated on the precoated plates at 37 °C and with 5% CO_2_ for 72 h. After this, the cell nuclei were stained with Hoechst 33342 dye (H3570, Invitrogen) and observations were made using the ZEISS Axio Observer fluorescence microscope (ZEISS, Oberkochen, Germany) and processed by ZEN software version 3.4 (blue edition). At each point, the syncytia were counted manually by two independent researchers.

## 4. Conclusions and Future Perspectives

The presented results suggest the successful development of a liposomal formulation of a decoy receptor intended for therapeutic use in combating early SARS-CoV-2 infection. The aim of this study was to conduct a phase I clinical trial with a novel drug candidate. Therefore, in future research, it is necessary to conduct preclinical in vivo studies with various animal models. We plan to conduct such studies in line with the requirements of the International Council for Harmonisation of Technical Requirements for Pharmaceuticals for Human Use (ICH) and the Organisation for Economic Co-operation and Development (OECD), as well as with recommendations by the European Medicines Agency (EMA) and the Food and Drug Administration (FDA). This in vivo study will include toxicity studies using rat and rabbit models followed by efficacy studies using the hamster model and a pharmacokinetic study on rats, since SARS-CoV-2 has been previously shown to efficiently replicate in the respiratory tract of these animals, invading the central nervous system and generating a cytokine storm resembling the same phenomenon in humans [97,98]. In these non-lethal models, the assessments carried out will be based on parameters such as body weight loss, histopathological changes, drug concentrations in the blood and the bronchoalveolar lavage, etc., in alignment with other animal studies on the development of COVID-19 therapeutics [99]. Similar approaches based on the interaction of a human recombinant soluble ACE2 fragment with S-RBD have been implemented in various anti-COVID-19 therapies. Their effectiveness, however, is limited by low blood concentrations and a short plasma half-life [15,100]. Creating a stable, inhalable carrier for ACE2-derived peptides is a promising approach, with this carrier acting at the site with the highest pathogen concentration. Inhaled liposomal formulations have been used in several studies on lung diseases, particularly on pulmonary fibrosis, lung infections, asthma, and lung cancer [101,102,103,104]. The main advantages of introducing liposome-based carriers include a reduction in the toxicity of the therapeutic agent, improvements in its pharmacokinetics and efficacy, and increased delivery, but features that do not affect the functioning of the biologically active compound itself, such as taste masking, may also be beneficial [104,105,106]. The major advantage of the proposed drug candidate compared to the already existing treatments described in the Section 1 is its very low sensitivity to the mutational variability of the RNA of the viral genome because it is based on the sequence of the human ACE2 derivative with an extremely low mutation rate. Extensive preclinical studies have demonstrated that this approach is successful against SARS-CoV-2 infection. However, half-life extension and effector function enhancement are recommended as directions for further development [107]. The introduction of this liposomal carrier is expected to significantly improve the stability of the peptide and improve its efficacy by allowing for the better exposition and increased visibility of the virus-liposomes conglomerates to the immunological system, which will be the subject of the follow-up functionality studies. The presented work is focused on the development of a carrier for the ACE2 derivative that can serve as a trap to immobilize the SARS-CoV-2 virus on the liposome surface. The same carrier can also be used against other viral infections in future. In fact, the multivalent character of the developed liposomes offers a strategy to enhance the targeting efficiency of various therapeutics [108,109]. The formulation obtained in the project is characterized by its nano-size (diameter below 100 nm), the 8–12-month stability of its physicochemical parameters, and its biological activity, i.e., the effective inhibition of a SARS-CoV-2 protein S–ACE2 interaction in a HEK293 cellular system. The aforementioned protocols can be applied during the development of different drug candidates, and the PEGylated liposomes demonstrated can serve as a platform for the immobilization and stabilization of various therapeutic molecules.

## 5. Patents

The authors declare pending patents no P.435921 and P.451191 (Patent Office of the Republic of Poland) and no. EP21889696.7 (European Patent Office).

## Figures and Tables

**Table 1:** Lipid composition of tested liposomal formulations during the development of anti-SARS-CoV-2 maleimide-functionalized therapeutic peptidoliposomes.

Lipid Concentration in Tested Formulations (mol%)												
Lipid	HSPC	CHOL	DSPC	DPPG	DOPG	POPG	EggPG	DSPE-PEG-1K	DSPE-PEG-2K	DSPE-PEG-2K-Mal	LysoPC	*n*
Formula #												
1	90	10	-	-	-	-	-	-	-	-	-	2
2	70	10	10	-	-	5	-	4.5	-	0.5	-	6
3	70	10	10	5	-	-	-	4.5	-	0.5	-	10
4	-	10	80	5	-	-	-	4.5	-	0.5	-	1
5	69	10	10	-	-	5	-	4.5	-	0.5	1	1
6	60	20	10	-	-	5	-	4.5	-	0.5	-	1
7	50	30	10	-	-	5		4.5	-	0.5		1
8	70	10	10	-	-	-	5	4.5	-	0.5	-	4
9	74.5	10	10	-	-	5	-	-	-	0.5	-	1
10	65	10	10	-	-	10	-	4.5	-	0.5	-	1
11	56	39	-	-	-	-	-	-	4.5	0.5	-	3
12	70	10	10	-	5	-	-	4.5	-	0.5	-	1
13	70	10	10	-	-	5	-	4	-	1	-	1
14	70	10	10	-	-	5	-	2.5	-	2.5	-	2
15	70	10	10	-	-	5	-	-	-	5	-	6
16	65	10	10			5	-	-	-	10	-	1
17	55	10	10	-	-	5	-	-	-	20	-	1
18	70	10	10	-	-	5	-	5	-	-	-	1
19	70	10	10	-	-	-	5	-	-	5	-	7
											Σ	51

Abbreviations: CHOL—cholesterol; DSPC—1,2-distearoyl-sn-glycero-3-phosphocholine; DSPE-PEG-1K—1,2-distearoyl-sn-glycero-3-phosphoethanolamine-N-[methoxy(polyethylene glycol)-1000]; DSPE-PEG-2K—1,2-distearoyl-sn-glycero-3-phosphoethanolamine-N-[methoxy(polyethylene glycol)-2000]; DSPE-PEG-2K-Mal—1,2-distearoyl-sn-glycero-3-phosphoethanolamine-N-[maleimide(polyethylene glycol)-2000]; DOPG—1,2-dioleoyl-sn-glycero-3-phospho-(1′rac-glycerol), DPPG—1,2-dipalmitoyl-sn-glycero-3-phospho-(1′rac-glycerol); EggPG—L-α-phosphatidylglycerol from egg; HSPC—hydrogenated soy L-α-phosphatidylcholine; LysoPC—1-stearoyl-sn-glycero-3-phosphocholine; POPG—1-palmitoyl-2-oleoyl-sn-glycero-3-phospho-(1′racglycerol).

**Table 2:** The average hydrodynamic diameter, polydispersity index (PDI), and zeta potential (ZP) of peptidoliposomes with phosphatidylcholine modifications synthesized with high-pressure homogenization (HPH) or with pressure extrusion techniques. For each formulation, *n* = 3.

	Formula #3	Formula #4	Formula #2	Formula #5
Calibration method	HPH	HPH	Extrusion	Extrusion
Diameter (nm)	59.46 ± 0.56	75.79 ± 1.05	79.07 ± 0.86	75.24 ± 0.11
PDI	0.134 ± 0.015	0.221 ± 0.009	0.067 ± 0.048	0.057 ± 0.007
ZP (mV)	−27.33 ± 0.82	−26.37 ± 0.54	−30.78 ± 1.1	−30.60 ± 0.73

**Table 3:** The average hydrodynamic diameter (nm), polydispersity index (PDI), and zeta potential (ZP) of peptidoliposomes with increasing concentrations of cholesterol. For each formulation, *n* = 3.

	Formula #2	Formula #6	Formula #7
Cholesterol (mol%)	10	20	30
Diameter (nm)	77.12 ± 1.29	83.02 ± 1.33	107.73 ± 1.68
PDI	0.067 ± 0.017	0.059 ± 0.004	0.177 ± 0.010
ZP (mV)	−28.97 ± 1.26	−31.13 ± 1.25	−30.53 ± 0.69

**Table 4:** The average hydrodynamic diameter (nm), polydispersity index (PDI), and zeta potential (ZP) of peptidoliposomes with modified PG content. POPG—palmitoyloleoylphosphatidylglycerol; DPPG—dipalmitoylphosphatidylglycerol; EggPG—Egg L-α-phosphatidylglycerol; DOPG—dioleoylphosphatidylglycerol. For each formulation *n* = 3.

	Formula #2	Formula #3	Formula #8	Formula #10	Formula #12
PG (mol%)	5	5	5	10	5
PG type	POPG	DPPG	EggPG	DPPG	DOPG
Diameter (nm)	83.32 ± 0.90	77.12 ± 1.29	83.40 ± 0.86	79.88 ± 0.81	80.21 ± 1.05
PDI	0.075 ± 0.008	0.069 ± 0.017	0.070 ± 0.014	0.071 ± 0.010	0.138 ± 0.004
ZP (mV)	−30.40 ± 0.41	−28.97 ± 1.26	−30.60 ± 0.16	−32.67 ± 0.57	−30.77 ± 1.68

**Table 5:** The average hydrodynamic diameter (nm), polydispersity index (PDI), and zeta potential (ZP) of peptidoliposomes with modified DSPE-PEG_2000_-Mal content. For each formulation, *n* = 3.

Formula #	DSPE-PEG-2K-Mal (mol%)	Diameter (nm)	PDI	ZP (mV)
2	0.5	81.02 ± 0.43	0.068 ± 0.008	−32.60 ± 0.94
13	1.0	81.04 ± 0.60	0.080 ± 0.006	−23.80 ± 2.25
14	2.5	85.26 ± 0.68	0.077 ± 0.015	−21.17 ± 1.99
15	5.0	88.07 ± 0.052	0.105 ± 0.018	−24.57 ± 1.56
16	10.0	84.02 ± 0.20	0.078 ± 0.009	−25.73 ± 0.50
17	20.0	77.46 ± 0.57	0.183 ± 0.014	−29.53 ± 1.21

**Table 6:** The average hydrodynamic diameter with the most prominent peak size, width at half height (Peak 1 size (nm), peak intensity (%), HH width (nm), respectively), the polydispersity index (PDI), and the zeta potential (ZP) with the leading peak value, area, and width at half height (Peak 1 ZP (mV), Peak Area (%) and HH Width (mV), respectively), measured in liposomes (Formula #19) manufactured using the high-pressure homogenization method or pressure extrusion method directly after preparation (day 0) and after 365 days of storage at 4 °C.

	Homogenized Liposomes		Extruded Liposomes	
Time (days)	0	365	0	365
Diameter (nm)	57.18 ± 0.41	66.96 ± 0.43	80.39 ± 0.71	82.56 ± 1.24
Peak 1 size (nm)	63.69	70.83	85.50	89.13
Peak intensity (%)	99.7	98.2	100.0	100.0
HH Width (nm)	23.00	24.62	21.68	25.33
PDI	0.131 ± 0.015	0.188 ± 0.014	0.55 ± 0.015	0.078 ± 0.021
ZP (mV)	−25.77 ± 0.49	−21.8 ± 1.49	−34.40 ± 1.41	−26.4 ± 0.1
Peak 1 ZP (mV)	−25.8	−29.8	−33.8	−26.4
Peak Area (%)	100.0	86.1	100.0	100.0
HH Width (mV)	9.53	9.63	9.82	8.46

## Data Availability

All data supporting the reported results are available in a repository of project’s leaders—Silesian Park of Medical Technology KardioMed Silesia and ACELLMED (contact: biuro@kmptm.pl; biuro@acellmed.pl). Access to the data is subject to approval and data sharing agreement due to the project’s data confidentiality (project no 2021/ABM/05/00002-00).

## Supplementary Materials

The supporting information can be downloaded at https://www.mdpi.com/article/10.3390/ijms26041629/s1.

## Appendix A

At each point right after peptidoliposome synthesis and whenever necessary, the preparation was sterilized. The sterilization process was optimized, and two methods were selected for that purpose—extrusion for preparation with small volumes (up to 10 mL) and filtration with the Steriflip vacuum filtration system (Millipore) for larger volumes. Neither of the sterilization methods led to any significant decreases in the lipid and peptide concentrations (Supplementary Material, Figure S3).
