# Supplementary material for: Development of Stable, Maleimide-Functionalized Peptidoliposomes Against SARS-CoV-2
Source: Int J Mol Sci. 2025 Feb 14;26(4):1629. doi: 10.3390/ijms26041629 (PMC11855074; doi:10.3390/ijms26041629)
Supplement: Supplementary file 1 [file ijms-26-01629-s001.zip › ijms-3341432-supplementary.pdf]

International Journal of Molecular Sciences

SUPPLEMENTARY MATERIAL

Development of Stable, Maleimide-Functionalized Peptidoliposomes Against SARS-CoV-2

Olga Michel [1,*], Aleksandra Kaczorowska [1,2], Lucyna Matusewicz [1], Kliwia Piórkowska [3], Marlena Golec [3], Wiktoria Fus [3], Kazimierz Kuliczkowski [3], Aleksander F. Sikorski [4,5] and Aleksander Czogalla [1,*]

1 Department of Cytobiochemistry, Faculty of Biotechnology, University of Wrocław, F. Joliot Curie 14a, 50-383 Wrocław, Poland; aleksandra.kaczorowska@pwr.edu.pl (A.K.); lucyna.matusewicz2@uwr.edu.pl (L.M.)
2 Department of Biomedical Engineering, Faculty of Fundamental Problems of Technology, Wrocław University of Science and Technology, Wybrzeże S. Wyspiańskiego 27, 50-370 Wrocław, Poland
3 Silesian Park of Medical Technology Kardio-Med Silesia, M. Curie-Skłodowskiej 10C, 41-800 Zabrze, Poland; k.piorkowska@kmptm.pl (K.P.); marlena.golec@gliwice.nio.gov.pl (M.G.); w.fus@kmptm.pl (W.F.); kazkul@wp.pl (K.K.)
4 Acellmed Ltd., M. Curie-Skłodowskiej 10C, 41-800 Zabrze, Poland; aleksander.sikorski@wssk.wroc.pl
5 Research and Development Center, Regional Specialist Hospital, Kamienskiego73a, 51-154 Wrocław, Poland
* Correspondence: olga.michel@umw.edu.pl (O.M.); aleksander.czogalla@uwr.edu.pl (A.C.); Tel.: +48-71-784-06-88 (O.M.); +48-71-375-63-56 (A.C.)

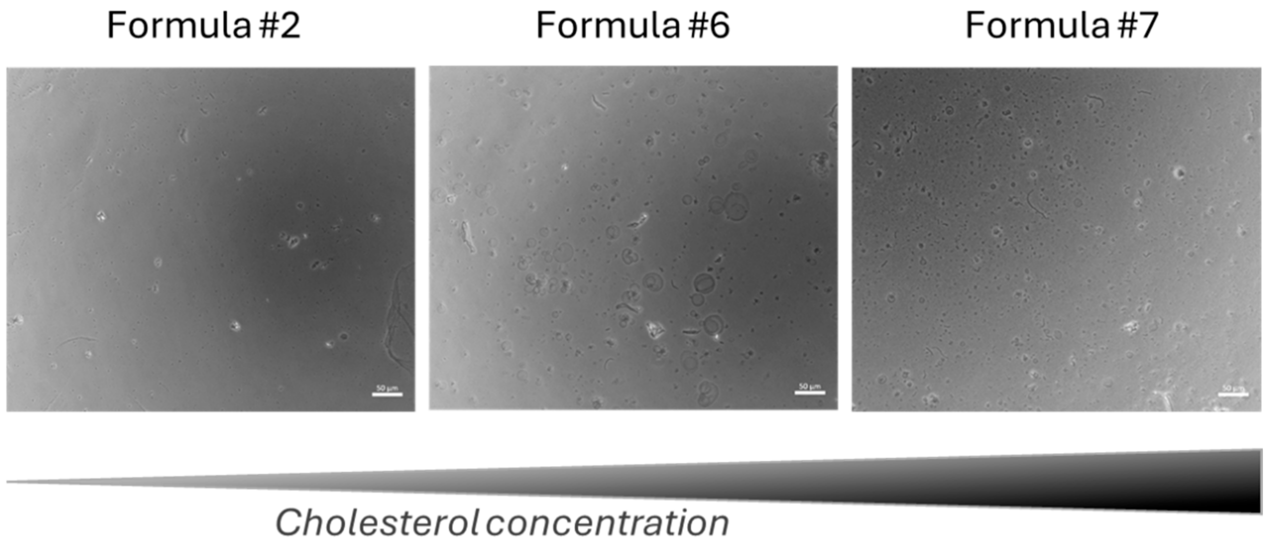

Figure S1. The formation of the inclusions in culture media in peptoliposomes with 10 mol% CHOL (Formula #2), 20 mol% CHOL (Formula #6) and 30 mol% CHOL (Formula #7) under the conditions of the accelerated aging assay (72 h at 37°C in the presence of culture medium). The scale bar is 50 μm.

International Journal of
Molecular Sciences

MDPI

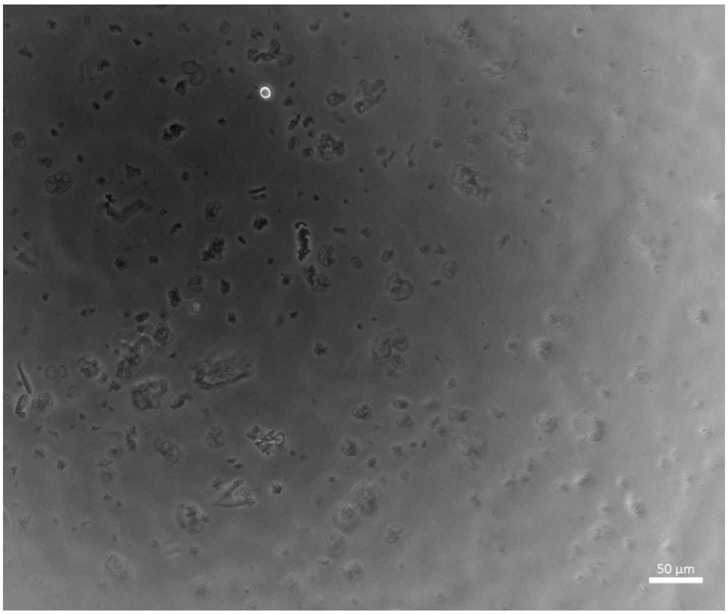

Figure S2. Microscopic photo of precipitates of the preparation containing 5 mol% DOPG (Formula #12) in the culture medium at a lipid concentration of 0.57 mg/ml. The scale bar is 50 µm.

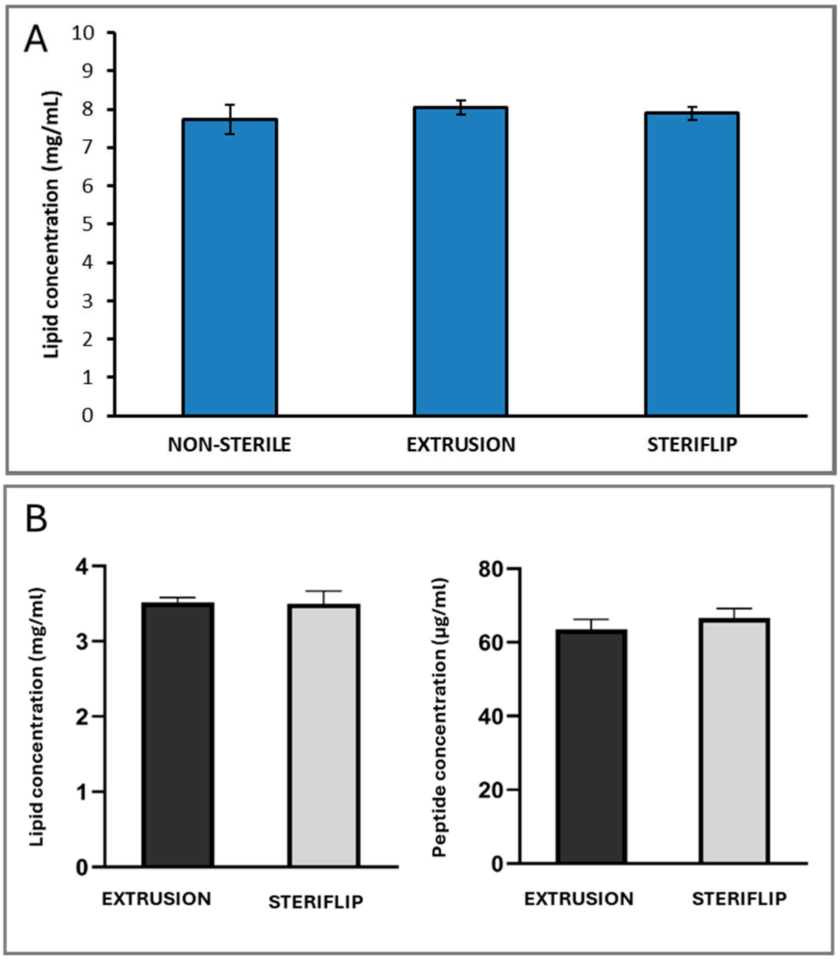

Figure S3. The effect of various sterilization techniques on the lipid and peptide concentration in peptoliposomes (Formula #3) calibrated with the extrusion technique (A) or with HPH (B) .

International Journal of
Molecular Sciences

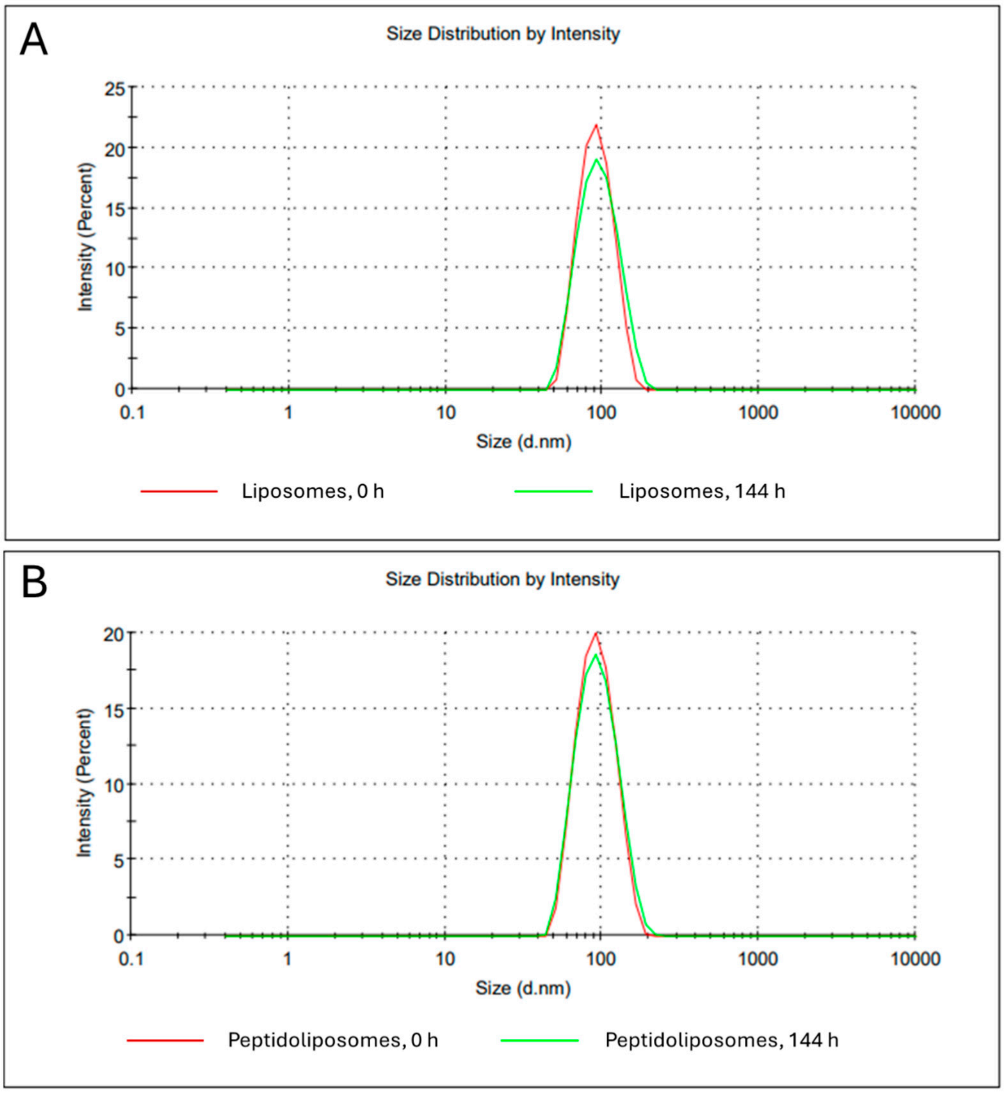

Figure S4. The average hydrodynamic diameters of liposomes (A) and peptidoliposomes (B) composed of Formula #19 in the presence of culture medium and FBS before (0h) and after 144 hours of incubation at 37°C (144h).
